# Fluoride Coatings on Magnesium Alloy Implants

**DOI:** 10.1155/2022/7636482

**Published:** 2022-03-07

**Authors:** ChuanYao Zhai, Chun Yu Dai, Xun Lv, Biying Shi, Yu Ru Li, Yifan Yang, Di Fan, Eui-Seok Lee, Yunhan Sun, Heng Bo Jiang

**Affiliations:** ^1^The Conversationalist Club, School of Stomatology, Shandong First Medical University & Shandong Academy of Medical Sciences, Tai'an, Shandong 271016, China; ^2^Department of Oral and Maxillofacial Surgery, Graduate School of Clinical Dentistry, Korea University, Seoul 08308, Republic of Korea

## Abstract

After several years of research and development, it has been reported that magnesium alloys can be used as degradable metals in some medical device applications. Over the years, fluoride coatings have received increasing research attention for improving the corrosion resistance of magnesium. In this paper, different methods for preparing fluoride coatings and the characteristics of these coatings are reported for the first time. The influence of the preparation conditions of fluoride coatings, including the magnesium substrate, voltage, and electrolyte, on the coatings is discussed. Various properties of magnesium fluoride coatings are also summarized, with an emphasis on corrosion resistance, mechanical properties, and biocompatibility. We screened experiments and papers that planned the application of magnesium fluoride coatings in living organisms. We have selected the literature with the aim of enhancing the performance of *in vivo* implants for reading and further detailed classification. The authors searched PubMed, SCOPUS, Web of Science, and other databases for 688 relevant papers published between 2005 and 2021, citing 105 of them. The selected time range is the last 16 years. Furthermore, this paper systematically discusses future prospects and challenges related to the application of magnesium fluoride coatings to medical products.

## 1. Introduction

Recently, with the rapid increase in the number of tissue injury repair procedures, metals have been widely used for the replacement and regeneration of injured tissues owing to their high mechanical properties [1]. Their common applications include scaffolds [2, 3], bone plates [4, 5], bone nails [6], wound closing devices [7], artificial joint prostheses [8], and guided tissue/bone regeneration membranes [9]. Nonbiodegradable metals used in traditional metal implants include stainless steel, titanium, and cobalt-chromium alloys [7, 10]. Despite their excellent biocompatibility and mechanical properties, they can cause inflammatory reactions because of the release of toxic ions, which often require secondary surgical removal [11, 12]. Moreover, the stress shielding effect of conventional bone implants often impedes healing because of the disparity in elastic modulus between the conventional metals and bone [10].

Fortunately, as a biodegradable metal, magnesium is preferred as a biologically essential trace element, with an elastic modulus similar to bone in fracture healing, eliminating the need for a secondary surgical removal [13]. The ideal clinical biodegradable metals must be perfectly suited for the injured tissue reconstruction in a biologically nontoxic precondition, providing absolute mechanical protection in the early stages and gradually degrading at an acceptable rate as the tissue heals [7]. Despite the developments in the research on magnesium alloys over the past decades, clinical studies on magnesium alloys can be traced back to 1878; at that time, Edward C. Huse first used magnesium wires to ligate blood vessels [14]. Nevertheless, the current bottleneck limiting the clinical application of magnesium is its extremely rapid degradation rate *in vivo*, which may result in the accumulation of local air pockets, an alkalinization effect, an osmotic pressure increase, and even a rapid decrease in the mechanical strength of the implants [2, 15]. Currently, there are two ways to control the degradation rate of magnesium: composition modification and alloy surface treatment. The properties of magnesium alloys can be influenced by changing the amount and percentage of alloying elements, like Al, Li, Ca, Y, Mn, Zn, Zr, and rare earth [16].

The ideal magnesium alloy coating has properties such as corrosion resistance, degradability, and biocompatibility for clinical applications [17]. Surface modifications are known to be classified according to the method of coating preparation, which include mechanical [18], physical [19], chemical [20], and biological or biomimetic. Chemical coating is formed by the reaction between the magnesium substrate and coating solution, which makes the chemical coating strongly bonded to the substrate [21]. Since the formation is based on chemical reactions, it is more sensitive to thermodynamics and kinetics [17]. Typical chemical coating techniques include chemical conversion, plasma electrolytic oxidation (PEO), thermal treatment, and electrodeposition [15]. Among them, chemical conversion is often used as a pretreatment [12]. PEO, also known as microarc oxidation (MAO), is the use of plasma arc discharge at the electrolyte/electrolyte interface to react with the electrolyte and sinter the substrate surface to form a coating [10]. The PEO layer is usually more stable than the chemical conversion layer, but its porous surface may lead to pit corrosion [12]. Fluoride coating, tightly bonded to the substrate and insoluble in water, is formed via chemical reactions between fluorine and magnesium by the specific methods listed above. The main degradation products, Mg^2^+ and low concentrations F^−^ have both been shown to enhance osteogenesis [5, 22]. Furthermore, F^−^ ions have been proved to have antibacterial properties in dentistry [23]. As a burgeoning coating, fluoride coating has been validated to improve the corrosion resistance of magnesium to a certain extent while also meeting the requirements of an ideal coating, such as self-degradability and biocompatibility, making it a promising coating [24–27].

Currently, there is only one review of immersed fluoride conversion coatings for medical magnesium alloys [28]; however, no review for fluoride coatings is available. Therefore, this paper reviews the advances in fluoride coatings for medical magnesium alloys, with the aim of discussing the pros and cons of existing fluoride coatings from the perspectives of preparation methods, coating structures and properties, and challenges and suggestions for further research.

## 2. Growth of Fluorinated Coatings

The fabrication of a dense, homogeneous, and biocompatible fluorinated coating on the surface of magnesium alloys by chemical transformation is a widely used treatment to enhance the corrosion resistance of magnesium alloys [29, 30], that is, HF acid immersion treatment.

**Figure 1:**
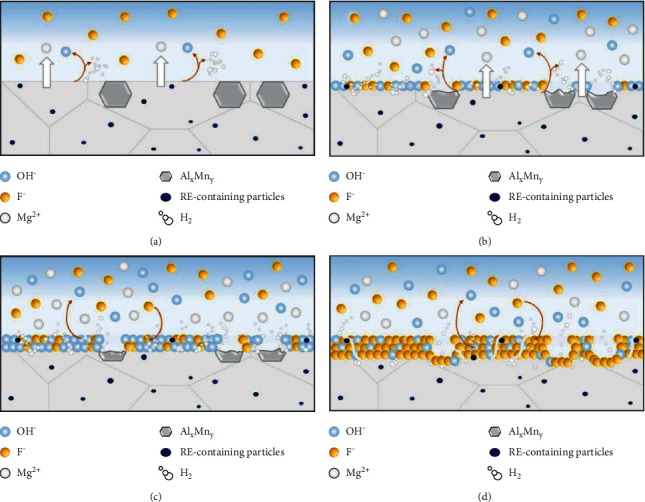
Mechanism of formation of the fluoride coating on the AZ31 magnesium alloy [31].

When the magnesium alloy is immersed in the HF solution, the magnesium alloy substrate is heavily corroded, producing Mg^2+^, H_2_, and OH^−^ (equations (1)–(3)). Subsequently, Mg^2+^ reacts with F^−^ and OH^−^ in the solution to form a compound on the surface of the substrate (equations (4)–(6)). Because Mg(OH)_2_ is extremely unstable under acidic conditions, it can undergo an exchange reaction (equation (7)), in which the OH^−^ within the Mg(OH)_2-x_F_x_ coating is replaced by F^−^ (Figure 1) [31]. The above reaction was also accelerated by increasing the concentration of HF in the conversion solution [31].

(1) $$\begin{array}{c} \mathrm{Mg}⟶Mg^{2+}+2e^{-} \end{array}$$

(2) $$\begin{array}{c} 2H^{+}+2e^{-}⟶H^{2}↑ \end{array}$$

(3) $$\begin{array}{c} 2H_{2}O+2e^{-}⟶2OH^{-}+H_{2}↑ \end{array}$$

(4) $$\begin{array}{c} Mg^{2+}+2F^{-}⟶\mathrm{Mg}F_{2} \end{array}$$

(5) $$\begin{array}{c} Mg^{2+}+2OH^{-}⟶\mathrm{Mg}{\left(\mathrm{OH}\right)}_{2} \end{array}$$

(6) $$\begin{array}{c} Mg^{2+}+XF^{-}+\left(2-X\right)OH^{-}⟶\mathrm{Mg}{\left(\mathrm{OH}\right)}_{2-x}F_{x} \end{array}$$

(7) $$\begin{array}{c} \mathrm{Mg}{\left(\mathrm{OH}\right)}_{2}\mathrm{+2}F^{-}⟶\mathrm{Mg}F_{2}+2OH^{-} \end{array}$$

Fluoride coatings have received more attention in recent years, particularly for methods such as immersion fluorination, microarc fluorination [32], and ultrasonic immersion fluorination [24] based on the composite fluoride coatings derived from the abovementioned methods, such as hydroxyapatite/magnesium fluoride composite coatings [33], fluoride-treated and sol-gel film composite coatings [34], and composite coatings with fluoride as a pretreatment, an electrolyte, or additives [35–41]; these composite and multilayer coatings are not discussed in detail in this paper because there are no strict standards for their conceptual classification.

## 3. Technology

Currently, there are four main technologies for preparation of magnesium-based magnesium fluoride coatings: anodic fluorination (AF), immersion fluorination (HF), ultrasonic immersion fluorination (UHF), and microarc fluorination (MAF).

### 3.1. Anodic Fluorination

Anodic fluorination is the replacement of the normal electrolyte with an electrolyte containing the element fluorine on the basis of anodic oxidation. Anodic fluorination uses the metal as an anode and forms a porous coating on the metal surface by means of electrolytic oxidation. After AF treatment, the surface of the sample forms a coral-like and shale-like surface morphology. Compared with untreated specimens, the treated specimens performed better in corrosion resistance. A better coating impedance effect appeared at relatively low voltages, which is consistent with the experimental expectations. In the low-voltage treatment group, the corrosion resistance of AF10, AF30, and AF20 showed a high to low level. 10 V treated samples showed the lowest current density and relatively high corrosion voltage. The thickness of the magnesium fluoride film increases with the increase of the voltage, reaching a peak at AF60. However, the bond between the coating and the substrate is not strong enough, and the coating tends to peel off as the coating thickness increases. Therefore, samples treated at 10 V have the best corrosion resistance [32].

In the same way as microarc fluoridation, the thickness and microstructure of the coating can be changed by varying the applied voltage under fixed electrolyte conditions. It is also more environmentally friendly and economical than microarc fluorination due to the lower applied voltage and lower electrolyte concentration [26].

### 3.2. Immersion Fluorination

Immersion is a popular technique for preparing coatings. The desired properties can be obtained by modifying the composition of the deposited layer. The traditional method of immersion fluorination involves immersing magnesium in a certain concentration of HF solution at a specific temperature for a certain amount of time before removing it [42].  Table 1 shows the characteristics of different HF-coated magnesium alloys prepared under various parameters. A thin fluoride film with MgF_2_ as the main component was formed on the surface of magnesium alloys [43]. The coating obtained by immersion fluorination can effectively decrease the degradation rate of magnesium alloys *in vivo*. Meanwhile, the coating showed good biocompatibility [44–46]. This method is appreciated for its simplicity, low cost, and easy control, while the coating formed is loose and porous and may easily peel off [47].

(8) $$\begin{array}{c} Mg_{\left(s\right)}+2HF_{\left(\mathrm{aq}\right)}⟶\mathrm{Mg}F_{2\left(s\right)}+2H_{2\left(g\right)},\;\Delta_{r}G^{{}^{\circ}}=-476.6\;\mathrm{kJ}/\mathrm{mol} \end{array}$$

(9) $$\begin{array}{c} Mg_{\left(s\right)}+2H_{2}O_{\left(l\right)}⟶\mathrm{Mg}{\left(\mathrm{OH}\right)}_{2\left(s\right)}+H_{2\left(g\right)},\;\Delta_{r}G^{{}^{\circ}}=-359.3\;\mathrm{kJ}/\mathrm{mol} \end{array}$$

(10) $$\begin{array}{c} \mathrm{Mg}{\left(\mathrm{OH}\right)}_{2\left(s\right)}+2H_{2}F_{\left(\mathrm{aq}\right)}⟶\mathrm{Mg}F_{2\left(s\right)}+2H_{2}O_{\left(l\right)},\;\Delta_{r}G^{{}^{\circ}}=-117.3\;\mathrm{kJ}/\mathrm{mol} \end{array}$$

**Figure 2:**
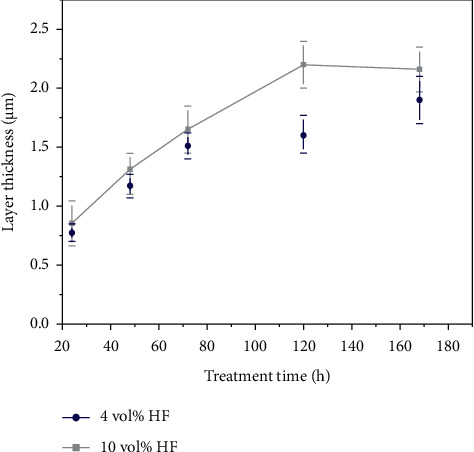
Variation of fluoride coating thickness on the AZ31 alloy as a function of treatment time [31].

Under different treatment conditions, the coating obtained may comprise Mg(OH)_2_, MgF_2_, and other substances, as shown in equations (8)–(10). Among them, the hydroxide in the coating was validated to have a negative impact on the corrosion resistance of the coating [54]. Since equations (8) and (9) have similar thermodynamic tendencies, it is assumed that both reactions will occur spontaneously and simultaneously when magnesium alloys are in contact with water and hydrofluoric acid. Thus, the rate of each reaction depends on the HF concentration [42]. When the acid concentration was too low, the Mg(OH)_2_ level in the coating was too high to lose the protective effect of the coating. According to equation (10), a higher concentration of HF converted the Mg(OH)_2_ generated during the treatment into MgF_2_. Barajas et al. [31] found that the layer obtained at 10 vol% HF (approximately 2.2 *μ*m) was thicker and had higher F content than that obtained at 4 vol% HF (approximately 1.9 *μ*m). The authors concluded that although the 10% HF-treated coating had more cracks, the higher F content of the coating might be a reasonable explanation for the corrosion resistance of the 10% HF-treated coating. However, an overly high concentration of HF may lead to thinning of the coating, which may be attributed to the fact that the dissolution rate of the magnesium substrate is faster than the generation rate of the conversion coating [55]. Additionally, treatment time has also been proven to affect the coating thickness [31, 44], which in turn affects the corrosion resistance of magnesium alloys by changing the probability of defects or “active spots” on the surface of the magnesium substrate, that is, the number of through-holes [56]. Usually, the thickness curve rises with treatment time and eventually flattens out (Figure 2) [31], but the formation rate of the coating gradually decreases, which may be related to the thickening of the coating that prevents the HF from reacting with the internal magnesium [57]. da Conceicao et al. [54] treated AZ31 at HF acid concentrations at a concentration gradient from 12 to 49 vol%. It was found that the coating thickness of the samples treated with high concentrations of HF acid was thinner and formed more slowly than those treated with low concentrations of HF acid, leading to a lower corrosion resistance due to the slower formation rate. The low concentration for a long time resulted in a high hydroxide content in the transformed layer, explaining the low corrosion resistance of the 12 vol% HF-treated coatings. Nevertheless, some studies have shown that magnesium alloys after alkaline pretreatment, which commonly refers to the reaction of magnesium alloys with high concentrations of NaOH to produce Mg(OH)_2_, can develop thicker MgF_2_ coatings than those without the alkaline pretreatment [3, 46, 50].

Furthermore, the crystalline phase on the alloy surface is also an essential factor affecting the formation of fluorinated magnesium coatings. Casanova et al. [58] analyzed the morphology of fluoride conversion coatings synthesized on Elektron 21 and AZ91D alloys. In a hydrofluoric acid (HF) solution, AZ91D and Elektron 21 alloys form MgF2 coatings, which provide good corrosion protection, where the presence and nature of different intermetallic phases play a pivotal role in its growth. The preferential dissolution of the reactive *β*-phase (Mg17Al12) in the AZ91D alloy promoted the growth of MgF2 coatings. Conversely, the microstructure of Elektron 21 alloys was more homogeneous, and the intermetallic compound Mg12(NdxGd1-x) phase remained stable, allowing the formation of continuous and homogeneous coatings. The microstructure of fluoride conversion coatings on AZ31 in relation to the degradation mechanism was studied by Barajas et al. The authors performed chemical conversion coating of AZ31 Mg alloy at 4 and 10% HF concentration with an immersion time of 24–168 hours [31]. During the conversion process, most of the metal particles in the *α*-Mg matrix on the surface of AZ31 were dissolved, but SEM observation revealed undissolved metal particles, and then EDX analysis revealed that the undissolved metal particles corresponded to rare earth-containing dispersions (La, Ce, and Nd). It may be due to the fact that Al is active under HF treatment conditions, and the preferred phase dissolved easily during the transformation process is AlxMny particles.

### 3.3. Ultrasonic Immersion Fluorination

One of the surface improvement methods that are both effective and environmentally friendly is HF. It enhances the corrosion and abrasion resistance of magnesium alloys by forming a thin and uniform fluoride coating that adheres to the alloy surface. However, this method is not applicable to clinical settings. Previous studies have shown that ultrasonic treatment of fluoride coatings can improve the corrosion resistance of Mg alloys and prepare denser and smoother coatings; this method is called ultrasonic immersion fluorination [24].

When immersed in an environment of 28 kHz ultrasound, the coatings of HF and UHF are identical in thickness and composition; however, UHF can reduce the porosity and cracks, exhibiting better corrosion resistance. The electrochemical tests showed that UHF had the highest electronic impedance and corrosion potential difference, as well as the lowest corrosion current density. Similarly, the mass loss test showed that the UHF-coated alloy exhibited a lower mass loss than the HF-coated and bare samples. Therefore, the ultrasonic treatment of magnesium alloy with fluoride coating is promising as a biomaterial in various medical applications [24].

Lellouche et al. reported on the antimicrobial and antibiofilm activities of nanosized magnesium fluoride (MgF2) nanoparticles (NPs) synthesized in ionic liquid using microwave chemistry [59]. Compounds nanosized MgF_2_ nanoparticles (MgF_2_NPs) by water-based synthesis of MgF_2_NPs using ultrasonic immersion. Ultrasonic chemical irradiation of aqueous solutions of ([Mg(Ac)_2_·(H_2_O)_4_]) containing hydrofluoric acid resulted in well-crystallized spherical MgF_2_NPs. Antimicrobial properties against two common bacteria (*Escherichia coli* and *Staphylococcus aureus*) were greatly improved. Using the ultrasonic chemical process described, the glass surface was coated, and the ultrasonically prepared magnesium fluoride crystals were shown to have an inhibitory effect on bacterial colonization within seven days.

### 3.4. MAF

The treatment of magnesium alloys in highly concentrated fluoride solutions using the microarc oxidation technique, also known as MAF, has the advantages of short treatment time and almost no crack formation. When MAF was performed, ammonium hydrogen fluoride and hydrofluoric acid were selected as the electrolytes. The higher the concentration of fluoride ions in the electrolyte, the more corrosion-resistant the fluoride coating; thus, a high concentration of HF (46%) is preferred as the electrolyte [26, 27]. In the electrolyte, a current is applied at a constant voltage for very short duration using magnesium alloy as the cathode and a graphite rod as the anode. According to the electrochemical and immersion tests, the best stability and corrosion resistance of the fluoride coating are achieved at 200 V, while too high voltage leads to the flaking of the coating [25].

The coatings prepared by MAF are dense and porous, with MgF_2_ as the main component; further, the corrosion resistance of the alloy is determined by factors such as pore size and surface roughness of the coating. Compared to HF and UHF, the coating structure of MAF is much denser and forms a coral-like structure on the surface of the alloys, resulting in a higher surface roughness that is proportional to the voltage [32]. Cell proliferation was significantly more enhanced in the treated samples than that in the bare Mg alloys [25, 60].

## 4. Properties

Magnesium alloys show promising biomedical applications owing to their biodegradability [61], with Young's modulus similar to that of bone, good biocompatibility, and osteogenesis. The ideal magnesium alloy implant maintains mechanical integrity during early implantation, provides absolute support, and eventually degrades as a bone defect or fracture repair without the requirement for secondary surgical removal [62]. In particular, magnesium, known to be one of the most essential substances in the human body, exists on human bone and soft tissue without obvious toxicity [25, 63–67] and is easily excreted in excess.

The extremely high rate of magnesium alloy degradation in humans severely limits their clinical applications. Based on the different properties of magnesium alloy fluoride coatings, the following is a comprehensive review of the effect of fluoride coatings on magnesium alloys, regardless of the limiting preparation techniques and experimental types (*in vivo*/*in vitro*). We hope to offer some valuable suggestions for improving the corrosion resistance, mechanical properties, substrate bonding strength, biocompatibility, bone integration and osteogenic activity, and antimicrobial properties of magnesium alloy fluoride coatings.

### 4.1. Corrosion Resistance

Poor corrosion resistance is a significant issue in magnesium implants. Electron microscopic fluoride films consist of fine particles, which improve problems such as voids and cracks on the metal surface. Thus, fluoride coatings can improve corrosion resistance by surface modification.

**Figure 3:**
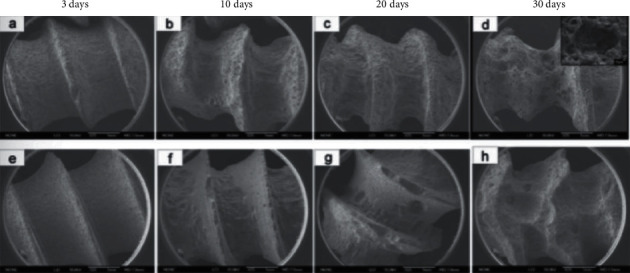
Scanning electron micrographs of the surface morphologies of uncoated MZZ screws (a–d) and MgF_2_-coated MZZ screws (e–h) after immersion in SBF for different durations [68].

The fluoride coating of magnesium alloys demonstrated excellent corrosion resistance in *in vitro* immersion experiments. Li et al. [68] made screws and tensile specimens from magnesium alloys as substrates and HF to obtain HF-coated magnesium alloy samples. After immersing the HF-coated and bare magnesium alloy samples in a simulated body fluid (HBSS), the immersed screw samples were subjected to scanning electron microscopy (SEM) (Figure 3) [68] and mass loss detection. The calculations showed that the corrosion rate of the coated screw samples was only one-quarter that of the uncoated samples because of the protection of the uniform and dense MgF_2_ coating. They also performed tensile tests and corrosion rate tests on tensile specimens after immersion, and the MgF_2_-coated samples showed a lower pitting corrosion rate than the bare samples, resulting in good mechanical properties even after one month of immersion.

In addition to HF-coated magnesium alloys, varying the parameters of different surface modification methods can also affect the corrosion resistance of magnesium alloys by changing the coating characteristics (Table 2).

The majority of the findings indicate that the electrical parameters have the greatest influence on the coating morphology and phase composition [72–80]. Heydarian et al. [70] used magnesium alloy as the substrate; the coating generated at a high voltage maintained corrosion resistance for 28 days without significant substrate corrosion. The study also reported that applying higher voltages to the coatings was more conducive to increasing the thickness of the coatings, and the further incorporation of fluoride in the coatings resulted in an increase in the MgF_2_ content in the inner layer of the coating, which contributed to the formation of coatings with stronger barrier properties.

Anodic polarization experiments were performed on untreated and 4.10 vol% HF immersion treated AZ31 magnesium alloy, and the electrochemical parameters were extracted as shown in Table 3 [31]; the majority of the coatings provided a scope of protection (Epit-Ecorr) to the metal substrate, and the fluoride coatings reduced the corrosion current density and enhanced the corrosion resistance of the alloy. Compared to previous studies [26], Dai et al. [32] used a low-voltage fluorination method to obtain coatings with controlled corrosion rates under safer conditions. This further confirms that MAF technology still has broad application prospects and research value in the use of magnesium alloy coatings. The above results show that the operating voltage has a significant influence on coating thickness.

**Figure 6:**
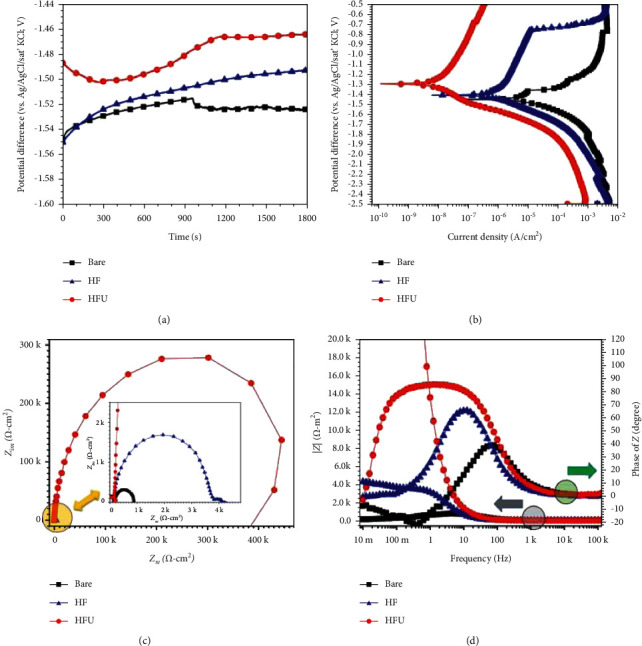
Electrochemical corrosion results. OCP (a), PDP (b), Nyquist (c), and bode (d) curves of the bare and HF- and HFU-coated AZ31 alloys [24].

Additionally, the preparation of fluoride coatings in an ultrasonic environment shows promise in the medical field. Sun et al. [24] used an AZ31 magnesium alloy for HF in a 28 kHz ultrasonic environment. The ultrasonic treatment of the coating allowed hydrogen to escape, resulting in a reduction in scratches and microporosity, as well as a significant increase in the corrosion resistance of the HFU-coating over the HF-coating. The electrochemical corrosion test results are represented in the curves shown in Figure 6 [24]. The HFU-coating had the lowest corrosion current density, highest corrosion potential, and highest electronic impedance, showing a noticeably higher corrosion resistance in the mass loss tests.

In summary, the formation conditions of the fluoride coating, such as voltage, current, and external conditions, determine its characterization and corrosion resistance.

### 4.2. Mechanical Property

Magnesium alloys must have mechanical properties to meet the bone-healing process in the human body during degradation. The mechanical properties of medical magnesium alloy implants are critical for the success of fracture fixation and cardiovascular surgery [63]. The more widely used metal implants, such as titanium, stainless steel, and cobalt-chromium alloys, require secondary surgical removal. The higher Young's modulus leads to a mechanical mismatch between the bone and implant, triggering a stress shielding phenomenon, which causes reabsorption of the surrounding bone [81]. Compared to polymeric materials, magnesium alloys have better mechanical properties and Young's modulus (44 GPa) closer to natural human bone (7–25 GPa) [82]. The protective effect of fluoride coatings on the mechanical properties (compressive, tensile, and bending properties) of biodegradable magnesium alloys in recent years is reviewed as follows [83].

Drynda et al. [84] demonstrated the protective effect of MgF_2_ coating by conducting four-point bending corrosion tests. They found MgF_2_-coated Mg-Ca alloys to be more suitable for biodegradable cardiovascular scaffolds than the currently available Mg alloys. Under constant load, the passivation of MgF_2_ coating occurred by forming Mg(OH)_2_ layer. Due to the small crack size (width <10 *μ*m; length <250 *μ*m), no large tensile stress is generated, and the Mg(OH)_2_ formed is relatively dense, which can separate magnesium alloy from the electrolyte and delay the corrosion process.

**Figure 7:**
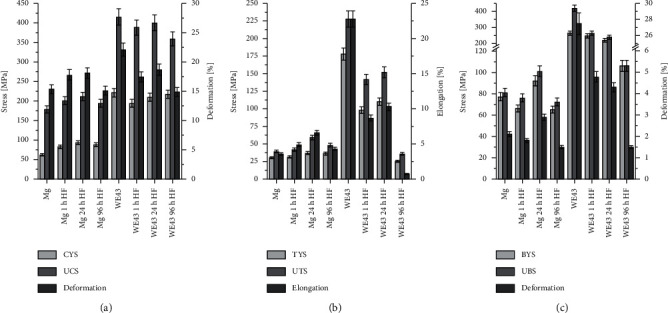
Mechanical properties of Mg and WE43 alloys: (a) compressive, (b) tensile, and (c) bending (compressive yield strength (CYS), ultimate compressive strength (UCS), tensile yield strength (TYS), ultimate tensile strength (UTS), bending yield strength (BYS), and ultimate bending strength (UBS)) [52].

Dvorsky et al. [52] measured the compressive, tensile, and flexural properties of different magnesium-based materials after HF, as shown in Figure 7 [52]. The mechanical properties of pure magnesium samples improved after fluorination, with the best mechanical properties achieved after 24 h of fluorination. The MgF_2_ coating formed after 1 h of immersion was thin and provided only a slight improvement in the mechanical properties; after 96 h of immersion, the coating was thicker, and brittleness increased. For the WE43 magnesium alloy, the exact opposite result was observed; a significant deterioration of the mechanical properties as the immersion time increased, which could be relevant to the inhomogeneous fluoride layer and YF3 phase. Therefore, the interaction between the substrate and the coating is also a vital factor affecting the mechanical properties.

**Figure 8:**
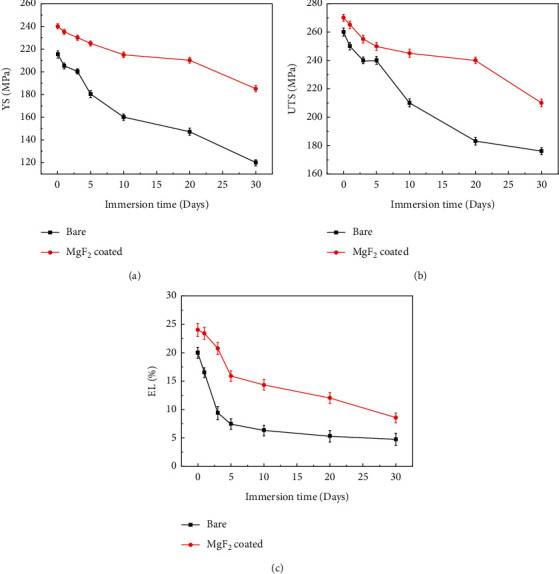
Bare and MgF_2_-coated MZZ during immersion for 30 days. (a) YS, (b) UTS, and (c) EL [68].

Li et al. [68] compared the mechanical properties of Mg-Zn-Zr (MZZ) alloy samples after immersion in SBF solution for various durations before and after the fluorination treatment, as shown in Figure 8 [68]. The yield strength (YS), ultimate tensile strength (UTS), and elongation (EL) of the fluorinated samples were much higher than those of the bare sample from day 3 to day 20 of the immersion, whereas the maximum corrosion rate (CRmax) of the coated samples was only approximately 50% that of the bare sample. These results indicate that the MgF_2_ coating can mitigate the effects of pitting corrosion on the magnesium matrix and contribute to maintaining preferable mechanical integrity.

### 4.3. Bonding with the Substrate

**Figure 9:**
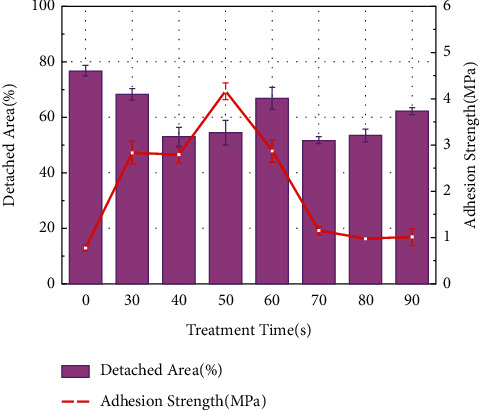
Average values of adhesive strength against the detached area for epoxy-coated aluminum samples with different Ti/Zr/V conversion treatment times [85].

**Figure 10:**
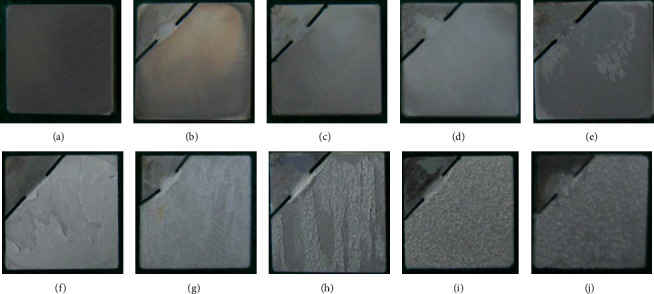
Optical observations of coated samples of (a) pure Mg, (b) AF10, (c) AF20, (d) AF30, (e) AF40, (f) AF50, (g) AF60, (h) AF70, (i) AF80, and (j) AF90 [32].

The prerequisite for a qualified coating to perform its excellent surface modification is a strong bond with the substrate. Chemical conversion methods are currently used for coating preparation, and the obtained coatings have a high bonding strength. Zhu et al. [85] compared the bonding strength of fluoride coatings with the substrate at different processing times, as shown in Figure 9 [85], and confirmed that the highest bonding strength was achieved at 50 s. Dai et al. [32] prepared fluoride coatings on magnesium alloy substrates. The surface morphologies of the generated coatings were compared at different voltages, and SEM images were obtained, as shown in Figure 10 [32]. Large areas of coating peeling appeared on the surface of the samples at voltages higher than 50 V. As stated in the study, the release of the plasma causes microporosity on the surface of the coating, leading to a coral-like appearance, which is required for the adhesion of the coating to the substrate. Excessively high voltages can roughen the coral-like structure, reducing adhesion. Furthermore, Heydarian et al. [70] used the PEO technique in their study to treat AZ91 magnesium alloy in an aluminate electrolyte. Comparing the magnesium fluoride coatings prepared at different voltages and observing the denseness and peeling of the coatings under SEM, it was confirmed that the voltage significantly affects the bond strength of the coating to the substrate. Consequently, it is possible to control the conditions during fluorination treatment to obtain fluoride coatings with better bond strength, which will have tremendous significance in clinical applications.

### 4.4. Biocompatibility

Magnesium alloy has good biocompatibility as a medical implant material [22, 68, 86–88]. Fluorine coating degrades and releases fluorine ions to surrounding tissues. A moderate amount of fluoride promotes teeth and bone growth and healing. In contrast, excessive fluoride in the body can lead to dental and skeletal fluorosis and affect the intellectual development of adolescents and the function of endocrine glands, damaging the gonads and other soft tissues such as the heart, liver, lungs, and kidneys. Therefore, while using fluoride as an implant coating, the advantages of fluoride in enhancing bone quality and accelerating calcification should be exploited as much as possible to avoid any harm to the body.

Extensive *in vivo* and *in vitro* experiments lay the foundation for the clinical application of magnesium fluoride implants. The MgF_2_-coated alloy improves its own corrosion resistance while maintaining the advantages of noncytotoxicity, favoring cell adhesion and proliferation, and not causing inflammation. *In vitro* cytotoxicity tests confirmed that the fluoride-coated AZ31B alloy is not toxic to human bone marrow mesenchymal stem cells (BMMSC) [49]. Jo et al. [89] performed an *in vitro* cellular response examination of preosteoblasts using cell proliferation assays and alkaline phosphatase (ALP) assays, indicating that hydroxyapatite coatings with MgF_2_ as an intermediate layer also enhanced the level of cell proliferation and differentiation. HA/MgF_2_-coated magnesium had higher corrosion resistance than bare magnesium and the bone-to-implant contact (BIC) ratio in the cortical bone region of the rabbit femur at 4 weeks after implantation. Durisin et al. [90] observed nonspecific inflammation and mucosal thickening in an *in vivo* study using a novel magnesium alloy scaffold placed in the paranasal sinus, confirming that the Mg-2 wt% Nd alloy scaffold coated with MgF_2_ has excellent biocompatibility while retaining functionality. These advantages make MgF_2_-coated magnesium alloys promising for long-term therapeutic applications in various medical fields. Regarding *in vivo* experiments, Constantin Carboneras et al. [91] observed the performance of nasal MgNd_2_ implants coated with MgF_2_ over 6 months and found slow histocompatible degradation of the implants without repeated bacterial infections. Drynda et al. [84] observed the biocompatibility of fluoride-coated magnesium-calcium alloy scaffolds in a subcutaneous mouse model, and none of the samples showed tissue inflammatory reactions or extensive proliferative effects compared to bare magnesium implants while improving corrosion resistance *in vivo*, suggesting that magnesium fluoride coating may be a good strategy to reduce biodegradation of magnesium-based alloys.

### 4.5. Bone Integration and Osteogenic Activity

Jiang et al. [22] prepared an MgF_2_ coating on an Mg-Zn-Zr alloy and implanted it into the femoral condyles of rabbits. The changes in corrosion resistance, biocompatibility, and osteogenic activity of the coated alloy were observed at the histological and micromorphological levels. It was concluded that the MgF_2_ coating was effective in reducing the rate of *in vivo* degradation of the Mg-Zn-Zr alloy. The bone tissue and mineral content gradually increased, demonstrating that the MgF_2_/Mg-Zn-Zr alloy promotes the formation of new bone on the alloy surface *in vivo*. Furthermore, the biological properties of the coating exhibited excellent biocompatibility and bioactivity.

**Figure 11:**
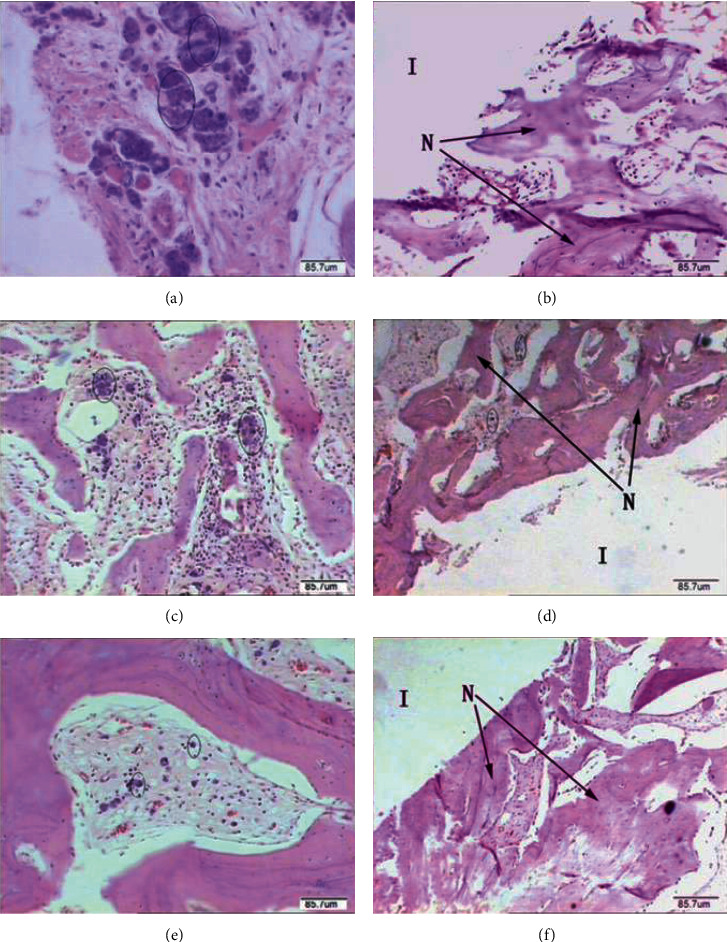
Histological photographs of the implant/bone interfaces around uncoated (a, b), Ca-P coating (c, d), and MgF_2_ coating (e, f) after 3 months after the operation (I: implant; N: newly formed trabecular bone; circle: magnesium granules) [92].

Sun et al. [92] conducted a similar work, coating degradable Mg-3Zn-0.8Zr cylinders with a Ca-P layer or an MgF_2_ layer; an uncoated Mg-3Zn-0.8zr alloy was used as a control group. Both specimens were implanted in the bone marrow of the white rabbits. During postoperative observation, SEM results showed a large number of cells, ample fibrillar collagen, and Ca-P products on the surface of the MgF_2_-coated implants. Also, micro-CT results revealed a slight decrease in volume (23.85%) and an increase in new bone volume (new bone volume fraction of 11.56% and tissue mineral density of 248.81 mg/cm^3^) in MgF_2_-coated implants after 3 months when compared to uncoated and Ca-P composite-coated implants. As the samples degraded, new bone trabeculae gradually formed, which was associated with a large number of active osteoblasts and osteocytes. The arrangement of newly formed bone trabeculae in the MgF_2_-coated samples (Figure 11(f)) [92] was much greater and more compact than the rest of the specimens. The bone trabeculae were well-structured and largely consistent with the original bone, which is in full accordance with Parfitt's study on the morphology of bone remodeling units [93].

**Figure 12:**
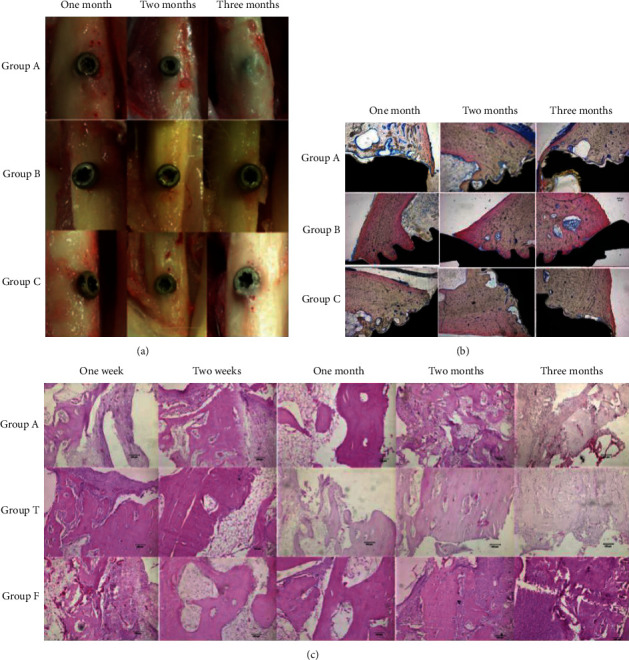
(a) Specimens of bone tissue reaction around implantations in different groups after various intervals of implantation. (b) Hard tissue section of the interface of implantation and bone in different groups after various intervals of implantation. (c) HE-stained sections around the implantations in different groups after various intervals of implantation. Group A, untreated AZ31 magnesium alloy screw; group T, titanium alloy screw; group F, AZ31 magnesium alloy screw coated with fluorine. These results showed that fluorine coating might promote the formation of new bone without obvious inflammatory reaction and fluorine-coated magnesium [6].

Sun et al. [6] implanted fluorine-coated AZ31B magnesium alloy screws in rabbit mandibles and femurs and discussed how fluorine coating enhances corrosion resistance and promotes bone formation of AZ31B magnesium alloy at the histological and immunohistochemical levels (Figure 12) [6]. Fluorine coating has been shown to enhance the corrosion resistance and bone formation of AZ31B magnesium alloy by upregulating type I collagen and BMP-2 expression (BMP-2 stimulates osteoclast differentiation and participates in bone tissue reconstruction) [94]. Nevertheless, due to the short observation time and complexity *in vivo*, further studies are required to clarify the exact mechanism by which degradation products affect osteogenesis.

### 4.6. Antibacterial Properties

When fluoride-coated magnesium alloys are used as surgical implants, the antibacterial requirements of the implants are strict due to the complexity of antibiotic treatment and wounds and the repetitive nature of surgery [95, 96]. There are various methods to improve the antimicrobial properties of the surface, such as reducing the generation of surface biofilm by coating properties, special coating space structure, and adding antimicrobial elements to the coating to change the environment or physiological function in which bacteria are located. The antimicrobial research of fluorinated coatings mainly focuses on the porous structure to change the surface PH antimicrobial and fluorine release antimicrobial.

Ren et al. [97] investigated the behavior of pure Mg and AZ31 alloys against *Escherichia coli* and *Staphylococcus aureus* with and without surface coatings. This paper focuses on the effects of surface pH, porosity, cracking, and coating density on surface antibacterial ability. The antimicrobial ability of pure Mg is high because of the very rapid rate of degradation, resulting in a significant increase in the surrounding pH to 10. Alkaline environments are not conducive to the growth and reproduction of *Escherichia coli* and *Staphylococcus aureus*. Robinson et al. [98] suggest that the degradable nature of Mg in physiological solutions causes a rapid increase in the Mg^2+^ concentration and the pH of the solution, with the latter supposedly being the cause of the bacterial inhibitory effect of Mg. Interestingly, if the Mg-based metal surface is covered with a porous layer, a relatively low degradation rate can not only be obtained but also acquire an antibacterial function to some extent. However, outside the fluorine-containing coatings of pure Mg and AZ31 alloys, the antimicrobial capacity is lost as the surface coating is too dense, slowing down the release of Mg^2+^ and leaving the pH of the surrounding tissue almost unchanged.

**Figure 13:**
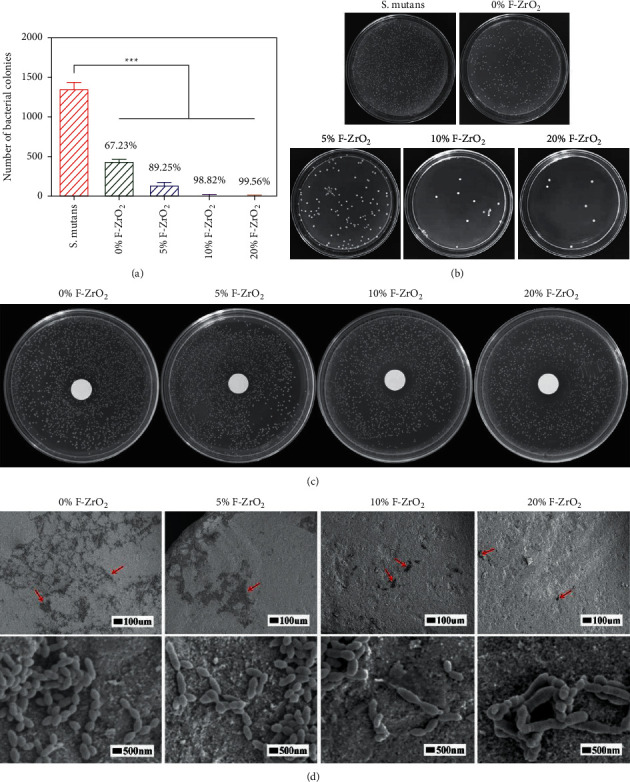
The results of antibacterial property of F-ZrO_2_ powders. (a) Numbers of bacterial colonies and the antibacterial rates of different groups of F-ZrO_2_ powders in CFUs counting. (b) Images of colonies of *S. mutans* after culturing with F-ZrO_2_ powders for 24 hours. (c) Images of the area of inhibition zones in the agar diffusion test (ADT). (d) SEM images of *S. mutans* on the specimens of F-ZrO_2_ disks (the red arrows differentiate the bacteria plaques on the surfaces of specimens) [99].

Due to the degradable nature of fluoride coatings, their fluoride-releasing properties are unquestionable. In oral studies, fluoride has been combined with other substances to release fluoride to improve its antimicrobial properties and prevent secondary caries. Although there are few studies related to the antimicrobial properties of MgF_2_ coatings associated with magnesium alloys, the study of fluoride releases to improve antimicrobial properties can provide a reference for the antimicrobial properties of fluorinated coatings. Zheng et al. [99] combined zirconia nanoparticles with fluorine (F-ZrO_2_) and investigated the effect of fluorine content on surface colonization. As shown in Figure 13 [99], the number of colonies decreased significantly with the addition of fluorine, indicating that F-ZrO_2_ has a significant antibacterial effect on *Streptococcus pyogenes*. Since the 20th century, fluoride has been shown to reduce the acid resistance of bacteria [100], and the application of fluoride-releasing materials has become a way to apply fluoride topically.

## 5. Challenges and Perspectives

Magnesium-based materials are limited in clinical applications because of the progressive decrease in mechanical properties caused by their fast degradation rate in the body fluid environment. Fluorination techniques are currently the most efficient and feasible solution for the surface modification of magnesium alloys.

Although many studies have been reported on the use of magnesium and its alloys, more extensive studies are still necessary to better evaluate the potential of fluoride coatings. The mechanical properties of magnesium materials, as well as their increased resistance to corrosion changes, must be thoroughly evaluated. Further optimization of corrosion-resistant fluoride coating technology is also a subject for further research. In addition, the effects of elemental fluorine entering human tissue fluids on biological organisms require extensive research data to support their safety.

## Figures and Tables

**Figure 4:**
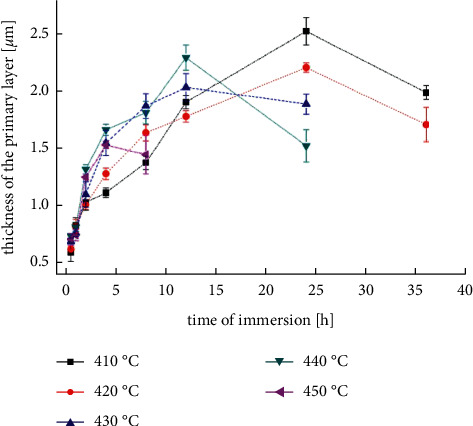
Coating thickness dependence on the coating time [69].

**Figure 5:**
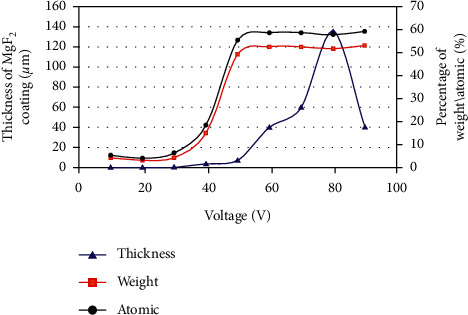
Variation of coating thickness and chemical compositions with processing voltage. In the figure, the blue triangle data point curve corresponds to the variation of coating thickness with the voltage [32].

**Table 1:** Characteristics of different HF-coated magnesium alloys prepared under various parameters.

Reference	Alloys	Treatment concentration	Treatment time	Thickness of the coatings	Special structure	Composition of coating	Special pretreatment
[48]	Mg-Zn alloys	HF 40 wt%	72 h	1-2 *μ*m	Tower-shaped pores	Mg, Zn, and F elements; MgF_2_ was not detected	—

[49]	AZ31B	HF 50 wt%	48 h	1.9 *μ*m	—	MgO and MgF_2_	—

[3]	Mg-Znp-Y-Nd alloys	HF 40% (v/v)	24 h	1.5–1.6 *μ*m	Smooth surface without pits	MgF_2_ and MgO	Treating in 5 M boiled NaOH for 3 h

[46]	LAE442	HF 40%	96 h	150–200 *μ*m	—	MgF_2_	Boiling in NaOH under slow stirring

[50]	Mg-Ca alloys	HF 40%	96 h	10–20 *μ*m	—	MgF_2_	Boiling in NaOH (*c* = 200 g/l) for 3 h under slow stirring

[44]	High-purity Mg	HF 40 wt%	24 h	3 *μ*m	—	MgF_2_	—
			48 h	3.5 *μ*m			
			96 h	4 *μ*m			

[51]	AZ61 particles	HF 40%	6 h	Nearly 3.82 *μ*m	Rough surface of particles with spots (uneven precipitates) scattered on the surface	MgF_2_	—
			12 h		—		
			24 h		Spots completely wrapping around the surface of spherical particles were observed		
			48 h		Significant deformation and cracking of the particles were observed		

[52]	WE43 (aerosolized particulate)	HF 40%	1, 24, and 96 h	—	Irregular, related to the YF_3_ phase between grains	MgF_2_ and YF_3_ phase	—
	HP Mg (aerosolized particulate)			3.4–3.8 *μ*m (maximum thickness was reached at 8 h)	Continuous uniformity (for 24 and 96 h instead of 1 h)	MgF_2_	

[31]	AZ31	HF 4 vol% or 10 vol%	24, 72, and 168 h	Shown in Figure 2 [31]	Cracks were observed	MgF_2_	—

[53]	Mg powder (Merck: CAS 7439-95-4); crystal powder of sucrose (C_12_H_22_O_11_: Merck: 1076531)	HF 48 wt%	15 h	1.4 *μ*m	—	MgF_2_ and MgO	—

**Table 2:** Characteristics of different HF-coated magnesium alloys prepared under various parameters.

Reference	Alloys	Treatment	Thickness of the coatings	Special structure	Composition of coating	*E*_corr_ (V)	*i*_corr_ (A/cm^2^)	The reference electrode and the electrolyte
[69]	AZ61	Treating by unconventional fluoride conversion in Na[BF_4_] molten salts at 410, 420, 430, 440, and 450°C for 0.5, 1, 2, 4, 8, 12, 24, and 36 h	Shown in Figure 4 [69].	No porosity or structural defects	Primary layer: Mg-F; secondary layer: Na[MgF3]	—	—	—

[24]	AZ31	Treating by ultrasonic immersion fluorination in HF 46% solution for 24 h	7.7 *μ*m	Nanocrystalline structure	MgF_2_	HFU: −1.298	HFU: 9.231 × 10^−7^	Reference electrode: Ag/AgCl/Sat-KCl (+197 mV). Electrolyte: HBSS

[27]	Pure Mg	Treating by microarc fluorination (MAF) in saturated NH_4_HF_2_ solution by constant voltages of 120, 160, 200, and 210 V for 3 min	MAF120: 2.5 *μ*m	MAF120: long-slot shape structure	MgF_2_	−1.573 (the pure Mg group is −1.842)	0.301 × 10^−6^ (the pure Mg group is 5.064 × 10^−6^)	Reference electrode: Ag/AgCl/Sat-KCl (+197 mV). Electrolyte: SBF
			MAF160: 3.5 *μ*m	MAF160: uniform and porous		−1.558	0.238 × 10^−6^	
			MAF200: 5.5 *μ*m	MAF200: uniform and porous		−1.547	0.187 × 10^−6^	

[26]	AZ31	Treating by plasma electrolytic fluorination in the pure NH_4_HF_2_ (150°C) by voltages of 100, 110, 120, 130, and 140 V for 30 s	PEF100: 1 and 3 *μ*m	PEF100, PEF110, and PEF120: a rough structure with a nonuniform texture. PEF130 and PEF140: porous and uniform structure	MgF_2_	−1.363 (the bare group is −1.543)	6.811 × 10^−6^ (the bare is 2.470 × 10^−5^)	Reference electrode: Ag/AgCl/Sat-KCl (+197 mV). Electrolyte: HBSS
			PEF110: 2.7 *μ*m			−1.403	6.498 × 10^−6^	
			PEF120: 5.6 *μ*m			−1.388	3.975 × 10^−6^	
			PEF130: 13.6 *μ*m			−1.358	8.533 × 10^−7^	
			PEF140: 13.9 *μ*m			−1.334	4.360 × 10^−6^	

[32]	Pure Mg	Treating by anodic fluorination (AF) at 0.1 mol/L NH_4_HF_2_ solution by direct current (CD) power supply at 10, 20, 30, 40, 50, 60, 70, 80, and 90 V for 3 min	Shown in Figure 5 [32]	AF10: a dot-like morphology. AF30 and AF40: a homogeneous matte-like appearance (as the voltage increased, the coral-like shape became coarser and shale-like)	MgF_2_	No specific data are mentioned in the article	AF10: 6.37 × 10^−6^ (the pure Mg group is 2.25 × 10^−5^)	Reference electrode: Ag/AgCl/Sat-KCl (+197 mV). Electrolyte: HBSS
							AF20: 4.13 × 10^−6^	
							AF30: 7.15 × 10^−6^	
							AF50: 2.61 × 10^−5^	
							AF60: 3.83 × 10^−5^	
							AF70: 8.55 × 10^−5^	
							AF80: 6.98 × 10^−5^	
							AF90: 7.72 × 10^−5^	

[25]	AZ31	Treating by microarc fluorination (MAF) anodized by constant voltage at 100, 150, 200, 250, and 300 V for 30 s in 46% HF solution	150 V: 0.5 *μ*m	The coral-like structure appeared	MgF_2_	−1.318 (the bare AZ31 group is −1.501)	0.228 × 10^−6^ (the bare AZ31 group is 342.4 × 10^−6^)	
			200 V: 0.6 *μ*m	The coral-like structure		−1.262	0.177 × 10^−6^	
			250 V: 0.7 *μ*m	The coral-like structure disappeared		−1.293	0.199 × 10^−6^	

[70]	AZ91	Treating by plasma electrolytic oxidation (PEO) at an aluminate-based electrolyte containing NaAlO_2_, NaF, and KOH at pH 12.20 at 32 ± 2°C for 10 min at two constant anodic voltages of 350 and 400 V using three different waveforms of unipolar, bipolar with 20% cathodic duty cycle and bipolar with 40% cathodic duty cycle for 10 min	The coatings are all thick on the outside and thin on the inside	Obvious microcracks and microporosity were observed on the surface. Double-layer structure of coating: porous outer layer and dense inner layer	MgO, MgAlO_4_, and MgF_2_	—	—	—
			Unipolar waveform: 4 *μ*m	Uniformly distributed pores were observed on the surface, showing regular circular holes with different sizes				
			Bipolar waveform: 15 *μ*m	Surface cavities with a crater-like morphology along with some granules of oxide were observed				

[71]	AZ31	Treating by potentiostatic polarization measurements using a potentiostat/Galvanostat 273 A at −1.4 V in 0.1M KF solution at room temperature	The inner layer: 300 nm	Compact	KMgF_3_, Mg(OH)_2_, and MgF_2_ are not detected	—	—	—
			The outer layer: 260 nm	Rough				

**Table 3:** Electrochemical parameters obtained from the anodic polarization curves [31].

Immersion time in HF (h)	4 vol% HF-treated sample				10 vol% HF-treated sample			
	Ecorr (V)	Epit (V)	Icorr (A/cm^2^)	Protection range (V)	Ecorr (V)	Epit (V)	Icorr (A/cm^2^)	Protection range (V)
0	−1.51	-	2.21 × 10–5	—	−1.51	—	2.21 × 10–5	—
24	−1.29	−1.14	1.70 × 10–7	0.15	−1.45	−1.19	1.19 × 10–7	0.26
48	−1.48	−1.26	5.84 × 10–7	0.22	−1.36	−1.11	1.69 × 10–7	0.25
72	−1.36	−1.19	2.28 × 10–7	0.17	−1.34	−1.21	1.23 × 10–7	0.13
168	−1.27	—	1.51 × 10–7	—	−1.24	−1.17	6.05 × 10–8	0.07

## Data Availability

All data, figures, and tables in this review paper are labeled with references.

## References

[1] Agarwal S., Curtin J., Duffy B., Jaiswal S. (2016). Biodegradable magnesium alloys for orthopaedic applications: a review on corrosion, biocompatibility and surface modifications. *Materials Science and Engineering: C*.

[2] Yu W., Zhao H., Ding Z. (2017). In vitro and in vivo evaluation of MgF2 coated AZ31 magnesium alloy porous scaffolds for bone regeneration. *Colloids and Surfaces B: Biointerfaces*.

[3] Wang P., Liu J., Shen S. (2019). In vitro and in vivo studies on two-step alkali-fluoride-treated Mg-Zn-Y-Nd alloy for vascular stent application: enhancement in corrosion resistance and biocompatibility. *ACS Biomaterials Science & Engineering*.

[4] Chaya A., Yoshizawa S., Verdelis K. (2015). In vivo study of magnesium plate and screw degradation and bone fracture healing. *Acta Biomaterialia*.

[5] Naujokat H., Ruff C. B., Klüter T., Seitz J.-M., Açil Y., Wiltfang J. (2020). Influence of surface modifications on the degradation of standard-sized magnesium plates and healing of mandibular osteotomies in miniature pigs. *International Journal of Oral and Maxillofacial Surgery*.

[6] Sun W., Zhang G., Tan L., Yang K., Ai H. (2016). The fluoride coated AZ31B magnesium alloy improves corrosion resistance and stimulates bone formation in rabbit model. *Materials Science and Engineering: C*.

[7] Zheng Y. F., Gu X. N., Witte F. (2014). Biodegradable metals. *Materials Science and Engineering: R: Reports*.

[8] Liu D., Ma Z., Zhang W., Huang B., Zhao H., Ren L. (2021). Superior antiwear biomimetic artificial joint based on high-entropy alloy coating on porous Ti6Al4V. *Tribology International*.

[9] Zhang H. Y., Jiang H. B., Kim J.-E., Zhang S., Kim K.-M., Kwon J.-S. (2020). Bioresorbable magnesium-reinforced PLA membrane for guided bone/tissue regeneration. *Journal of the Mechanical Behavior of Biomedical Materials*.

[10] Tian P., Liu X. (2015). Surface modification of biodegradable magnesium and its alloys for biomedical applications. *Regenerative Biomaterials*.

[11] Kim J.-H., Kook M.-S., Ryu S.-Y., Oh H.-K., Park H.-J. (2008). A simple technique for the treatment of inferior orbital blow-out fracture: a transantral approach, open reduction, and internal fixation with miniplate and screws. *Journal of Oral and Maxillofacial Surgery*.

[12] Hornberger H., Virtanen S., Boccaccini A. R. (2012). Biomedical coatings on magnesium alloys-a review. *Acta Biomaterialia*.

[13] Feeney K. A., Hansen L. L., Putker M. (2016). Daily magnesium fluxes regulate cellular timekeeping and energy balance. *Nature*.

[14] Witte F. (2010). The history of biodegradable magnesium implants: a review. *Acta Biomaterialia*.

[15] Staiger M. P., Pietak A. M., Huadmai J., Dias G. (2006). Magnesium and its alloys as orthopedic biomaterials: a review. *Biomaterials*.

[16] Prasad S. V. S., Prasad S. B., Verma K., Mishra R. K., Kumar V., Singh S. (2021). The role and significance of magnesium in modern day research-a review. *Journal of Magnesium and Alloys*.

[17] Yin Z.-Z., Qi W.-C., Zeng R.-C. (2020). Advances in coatings on biodegradable magnesium alloys. *Journal of Magnesium and Alloys*.

[18] Xiong X., Yang Y., Li J. (2019). Research on the microstructure and properties of a multi-pass friction stir processed 6061Al coating for AZ31 Mg alloy. *Journal of Magnesium and Alloys*.

[19] Guo Y., Zhang Y., Li Z. (2018). Microstructure and properties of in-situ synthesized ZrC-Al3Zr reinforced composite coating on AZ91D magnesium alloy by laser cladding. *Surface and Coatings Technology*.

[20] Chen X.-B., Yang H.-Y., Abbott T. B., Easton M. A., Birbilis N. (2013). Corrosion protection of magnesium and its alloys by metal phosphate conversion coatings. *Surface Engineering*.

[21] Gu X. N., Li N., Zhou W. R. (2011). Corrosion resistance and surface biocompatibility of a microarc oxidation coating on a Mg-Ca alloy. *Acta Biomaterialia*.

[22] Jiang H., Wang J., Chen M., Liu D. (2017). Biological activity evaluation of magnesium fluoride coated Mg-Zn-Zr alloy in vivo. *Materials Science and Engineering: C*.

[23] Ishiguro T., Mayanagi G., Azumi M. (2019). Sodium fluoride and silver diamine fluoride-coated tooth surfaces inhibit bacterial acid production at the bacteria/tooth interface. *Journal of Dentistry*.

[24] Sun J., Jin S., Zhao B. C. (2019). Enhanced corrosion resistance of biodegradable Mg alloys via ultrasonically treated fluoride coating. *Surface Topography: Metrology and Properties*.

[25] Sun L., Zhao B. C., Wang T. (2020). Surface characterization and corrosion resistance of biomedical AZ31 Mg alloy treated by microarc fluorination. *Scanning*.

[26] Jiang H. B., Wu G., Lee S.-B., Kim K.-M. (2017). Achieving controllable degradation of a biomedical magnesium alloy by anodizing in molten ammonium bifluoride. *Surface and Coatings Technology*.

[27] Jiang H. B., Kim Y. K., Ji J. H., Park I. S., Bae T. S., Lee M. H. (2014). Surface modification of anodized Mg in ammonium hydrogen fluoride by various voltages. *Surface and Coatings Technology*.

[28] da Conceição T. F., Scharnagl N. (2015). Fluoride conversion coatings for magnesium and its alloys for the biological environment. *Surface Modification of Magnesium and its Alloys for Biomedical Applications*.

[29] Carboneras M., García-Alonso M. C., Escudero M. L. (2011). Biodegradation kinetics of modified magnesium-based materials in cell culture medium. *Corrosion Science*.

[30] Chiu K. Y., Wong M. H., Cheng F. T., Man H. C. (2007). Characterization and corrosion studies of fluoride conversion coating on degradable Mg implants. *Surface and Coatings Technology*.

[31] Barajas J. D., Joya J. C., Durán K. S., Hernández-Barrios C. A., Coy A. E., Viejo F. (2019). Relationship between microstructure and formation-biodegradation mechanism of fluoride conversion coatings synthesised on the AZ31 magnesium alloy. *Surface and Coatings Technology*.

[32] Dai C. Y., Gao X., Zhai C. (2021). Corrosion evaluation of pure Mg coated by fluorination in 0.1 M fluoride electrolyte. *Scanning*.

[33] Bakhsheshi-Rad H. R., Idris M. H., Abdul-Kadir M. R. (2013). Synthesis and in vitro degradation evaluation of the nano-HA/MgF_2_ and DCPD/MgF_2_ composite coating on biodegradable Mg-Ca-Zn alloy. *Surface and Coatings Technology*.

[34] Hu J. Y., Li Q., Zhong X. K., Luo F. (2010). Fluoride treatment and sol film composite technology for AZ91D magnesium alloy. *Transactions of the IMF*.

[35] Assadian M., Jafari H., Ghaffari Shahri S. M., Idris M. H., Almasi D. (2016). Topography, wetting, and corrosion responses of electrodeposited hydroxyapatite and fluoridated hydroxyapatite on magnesium. *Bio-Medical Materials and Engineering*.

[36] Chen Q., Zheng Y., Dong S., Chen X.-B., Dong J. (2021). Effects of fluoride ions as electrolyte additives for a PEO/Ni-P composite coating onto Mg alloy AZ31B. *Surface and Coatings Technology*.

[37] da Conceicao T. F., Scharnagl N., Dietzel W., Hoeche D., Kainer K. U. (2011). Study on the interface of PVDF coatings and HF-treated AZ31 magnesium alloy: determination of interfacial interactions and reactions with self-healing properties. *Corrosion Science*.

[38] Dong H. R., Ma Y., Wang S., An L. Y., Hao Y. (2018). Effect of potassium fluoride on growth and microstructure of MAO coatings on AZ91D magnesium alloys. *Rare Metal Materials and Engineering*.

[39] Fu L. X., Yang Y. X., Zhang L. L., Wu Y. Z., Liang J., Cao B. C. (2019). Preparation and characterization of fluoride-incorporated plasma electrolytic oxidation coatings on the AZ31 magnesium alloy. *Coatings*.

[40] Guo H. F., An M. Z. (2005). Growth of ceramic coatings on AZ91D magnesium alloys by micro-arc oxidation in aluminate–fluoride solutions and evaluation of corrosion resistance. *Applied Surface Science*.

[41] Hatami M., Yeganeh M., Keyvani A., Saremi M., Naderi R. (2017). Electrochemical behavior of polypyrrole-coated AZ31 alloy modified by fluoride anions. *Journal of Solid State Electrochemistry*.

[42] da Conceição T. F., Scharnagl N., Narayanan T. S. N. S., Park I.-S., Lee M.-H. (2015). 1-fluoride conversion coatings for magnesium and its alloys for the biological environment. *Surface Modification of Magnesium and its Alloys for Biomedical Applications*.

[43] Dvorsky D., Kubasek J., Vojtech D. (2018). A new approach in the preparation of biodegradable Mg-MgF_2_ composites with tailored corrosion and mechanical properties by powder metallurgy. *Materials Letters*.

[44] Lou J., Sun Y., Chen Y. (2021). Effects of MgF_2_ coating on the biodegradation and biological properties of magnesium. *Surface and Coatings Technology*.

[45] Khan N. A., Shin S., Jhung S. H. (2020). Cu_2_O-incorporated MAF-6-derived highly porous carbons for the adsorptive denitrogenation of liquid fuel. *Chemical Engineering Journal*.

[46] Witte F., Fischer J., Nellesen J. (2010). In vivo corrosion and corrosion protection of magnesium alloy LAE442. *Acta Biomaterialia*.

[47] Bommala V. K., Krishna M. G., Rao C. T. (2019). Magnesium matrix composites for biomedical applications: a review. *Journal of Magnesium and Alloys*.

[48] Chen Y., Song Y., Zhang S. X. (2011). Effect of fluoride coating on in vitro dynamic degradation of Mg-Zn alloy. *Materials Letters*.

[49] Yan T., Tan L., Zhang B., Yang K. (2014). Fluoride conversion coating on biodegradable AZ31B magnesium alloy. *Journal of Materials Science & Technology*.

[50] Drynda A., Seibt J., Hassel T., Bach F. W., Peuster M. (2013). Biocompatibility of fluoride-coated magnesium-calcium alloys with optimized degradation kinetics in a subcutaneous mouse model. *Journal of Biomedical Materials Research Part A*.

[51] Gao C., Zeng Z., Peng S., Tan W., Shuai C. (2021). A continuous MgF_2_ network structure encapsulated Mg alloy prepared by selective laser melting for enhanced biodegradation resistance. *Advanced Engineering Materials*.

[52] Dvorsky D., Kubasek J., Jablonska E., Kaufmanova J., Vojtech D. (2020). Mechanical, corrosion and biological properties of advanced biodegradable Mg–MgF_2_ and WE43-MgF_2_ composite materials prepared by spark plasma sintering. *Journal of Alloys and Compounds*.

[53] Toghyani S., Khodaei M., Razavi M. (2020). Magnesium scaffolds with two novel biomimetic designs and MgF_2_ coating for bone tissue engineering. *Surface and Coatings Technology*.

[54] da Conceicao T. F., Scharnagl N., Blawert C., Dietzel W., Kainer K. U. (2010). Surface modification of magnesium alloy AZ31 by hydrofluoric acid treatment and its effect on the corrosion behaviour. *Thin Solid Films*.

[55] Bakhsheshi-Rad H. R., Idris M. H., Kadir M. R. A., Daroonparvar M. (2013). Effect of fluoride treatment on corrosion behavior of Mg-Ca binary alloy for implant application. *Transactions of Nonferrous Metals Society of China*.

[56] Song G.-L., Shi Z. (2014). Corrosion mechanism and evaluation of anodized magnesium alloys. *Corrosion Science*.

[57] Eshed M., Lellouche J., Banin E., Gedanken A. (2013). MgF_2_ nanoparticle-coated teeth inhibit Streptococcus mutans biofilm formation on a tooth model. *Journal of Materials Chemistry B*.

[58] Casanova P. Y., Jaimes K. J., Parada N. J. (2013). Synthesis and evaluation of MgF_2_ coatings by chemical conversion on magnesium alloys for producing biodegradable orthopedic implants of temporary use. *Journal of Physics: Conference Series*.

[59] Lellouche J., Friedman A., Lellouche J.-P., Gedanken A., Banin E. (2012). Improved antibacterial and antibiofilm activity of magnesium fluoride nanoparticles obtained by water-based ultrasound chemistry. *Nanomedicine: Nanotechnology, Biology and Medicine*.

[60] Gao X. Z., Dai C. Y., Jia Q. (2021). In vivo corrosion behavior of biodegradable magnesium alloy by MAF treatment. *Scanning*.

[61] Zhang S., Zhang X., Zhao C. (2010). Research on an Mg-Zn alloy as a degradable biomaterial. *Acta Biomaterialia*.

[62] Yang Y., He C., Dianyu E. (2020). Mg bone implant: features, developments and perspectives. *Materials & Design*.

[63] Rahman M., Li Y., Wen C. (2020). HA coating on Mg alloys for biomedical applications: a review. *Journal of Magnesium and Alloys*.

[64] Liu H., Li D., Zhang Y., Li M. (2018). Inflammation, mesenchymal stem cells and bone regeneration. *Histochemistry and Cell Biology*.

[65] Dou Y., Cai S., Ye X. (2013). 45S5 bioactive glass-ceramic coated AZ31 magnesium alloy with improved corrosion resistance. *Surface and Coatings Technology*.

[66] Witte F., Hort N., Vogt C. (2008). Degradable biomaterials based on magnesium corrosion. *Current Opinion in Solid State & Materials Science*.

[67] Ling L., Cai S., Li Q., Sun J., Bao X., Xu G. (2021). Recent advances in hydrothermal modification of calcium phosphorus coating on magnesium alloy. *Journal of Magnesium and Alloys*.

[68] Li Z., Shizhao S., Chen M., Fahlman B. D., Debao Liu L., Bi H. (2017). In vitro and in vivo corrosion, mechanical properties and biocompatibility evaluation of MgF_2_-coated Mg-Zn-Zr alloy as cancellous screws. *Materials Science and Engineering: C*.

[69] Drábiková J., Fintová S., Ptáček P. (2020). Structure and growth kinetic of unconventional fluoride conversion coating prepared on wrought AZ61 magnesium alloy. *Surface and Coatings Technology*.

[70] Heydarian A., Atapour M., Hakimizad A., Raeissi K. (2020). The effects of anodic amplitude and waveform of applied voltage on characterization and corrosion performance of the coatings grown by plasma electrolytic oxidation on AZ91 Mg alloy from an aluminate bath. *Surface and Coatings Technology*.

[71] Wu L., Dong J., Ke W. (2013). Potentiostatic deposition process of fluoride conversion film on AZ31 magnesium alloy in 0.1M KF solution. *Electrochimica Acta*.

[72] Hakimizad A., Raeissi K., Golozar M. A., Lu X., Blawert C., Zheludkevich M. L. (2017). The effect of pulse waveforms on surface morphology, composition and corrosion behavior of Al_2_ O_3_ and Al_2_O_3_/TiO_2_ nano-composite PEO coatings on 7075 aluminum alloy. *Surface and Coatings Technology*.

[73] Sreekanth D., Rameshbabu N., Venkateswarlu K. (2012). Effect of various additives on morphology and corrosion behavior of ceramic coatings developed on AZ31 magnesium alloy by plasma electrolytic oxidation. *Ceramics International*.

[74] Li X., Liu X., Luan B. L. (2011). Corrosion and wear properties of PEO coatings formed on AM60B alloy in NaAlO_2_ electrolytes. *Applied Surface Science*.

[75] Hussein R. O., Zhang P., Nie X., Xia Y., Northwood D. O. (2011). The effect of current mode and discharge type on the corrosion resistance of plasma electrolytic oxidation (PEO) coated magnesium alloy AJ62. *Surface and Coatings Technology*.

[76] Lv G.-H., Chen H., Wang X.-Q. (2010). Effect of additives on structure and corrosion resistance of plasma electrolytic oxidation coatings on AZ91D magnesium alloy in phosphate based electrolyte. *Surface and Coatings Technology*.

[77] Jiang B. L., Wang Y. M. (2010). Plasma electrolytic oxidation treatment of aluminium and titanium alloys. *Surface Engineering of Light Alloys*.

[78] Cakmak E., Tekin K. C., Malayoglu U., Shrestha S. (2010). The effect of substrate composition on the electrochemical and mechanical properties of PEO coatings on Mg alloys. *Surface and Coatings Technology*.

[79] Lv G.-H., Chen H., Gu W.-C. (2008). Effects of current frequency on the structural characteristics and corrosion property of ceramic coatings formed on magnesium alloy by PEO technology. *Journal of Materials Processing Technology*.

[80] Blawert C., Heitmann V., Dietzel W., Nykyforchyn H. M., Klapkiv M. D. (2007). Influence of electrolyte on corrosion properties of plasma electrolytic conversion coated magnesium alloys. *Surface and Coatings Technology*.

[81] Jochem Nagels M., Marie¨lle Stokdijk PhD., Piet M., Rozing M. D. (2003). Stress shielding and bone resorption in shoulder arthroplasty. *Journal of Shoulder and Elbow Surgery*.

[82] Adekanmbi I., Mosher C. Z., Lu H. H., Riehle M., Kubba H., Tanner K. E. (2017). Mechanical behaviour of biodegradable AZ31 magnesium alloy after long term in vitro degradation. *Materials Science and Engineering: C*.

[83] Zhao Y., Wu G., Jiang J., Wong H. M., Yeung K. W. K., Chu P. K. (2012). Improved corrosion resistance and cytocompatibility of magnesium alloy by two-stage cooling in thermal treatment. *Corrosion Science*.

[84] Drynda A., Hassel T., Hoehn R., Perz A., Bach F. W., Peuster M. (2010). Development and biocompatibility of a novel corrodible fluoride-coated magnesium-calcium alloy with improved degradation kinetics and adequate mechanical properties for cardiovascular applications. *Journal of Biomedical Materials Research Part A*.

[85] Zhu W., Li W., Mu S., Yang Y., Zuo X. (2016). The adhesion performance of epoxy coating on AA6063 treated in Ti/Zr/V based solution. *Applied Surface Science*.

[86] Zhang Z.-Q., Yang Y.-X., Li J.-A., Zeng R.-C., Guan S.-K. (2021). Advances in coatings on magnesium alloys for cardiovascular stents-a review. *Bioactive Materials*.

[87] Zhang Y., Chen K., Liu H. (2020). A study of a biodegradable braided Mg stent for biliary reconstruction. *Journal of Materials Science*.

[88] Li L., Zhang M., Li Y., Zhao J., Qin L., Lai Y. (2017). Corrosion and biocompatibility improvement of magnesium-based alloys as bone implant materials: a review. *Regenerative Biomaterials*.

[89] Jo J.-H., Kang B.-G., Shin K.-S. (2011). Hydroxyapatite coating on magnesium with MgF_2_ interlayer for enhanced corrosion resistance and biocompatibility. *Journal of Materials Science: Materials in Medicine*.

[90] Durisin M., Reifenrath J., Weber C. M. (2017). Biodegradable nasal stents (MgF_2_ -coated Mg-2 wt %Nd alloy)-a long-term in vivo study. *Journal of Biomedical Materials Research. Part B, Applied Biomaterials*.

[91] Carboneras M., Iglesias C., Pérez-Maceda B. T. (2011). Corrosion behaviour and in vitro/in vivo biocompatibility of surface-modified AZ31 alloy. *Revista De Metalurgia*.

[92] Sun J. e., Wang J., Jiang H., Chen M., Bi Y., Liu D. (2013). In vivo comparative property study of the bioactivity of coated Mg-3Zn-0.8Zr alloy. *Materials Science and Engineering: C*.

[93] Parfitt A. M. (1994). Osteonal and hemi-osteonal remodeling: the spatial and temporal framework for signal traffic in adult human bone. *Journal of Cellular Biochemistry*.

[94] Rosen V. (2009). BMP2 signaling in bone development and repair. *Cytokine & Growth Factor Reviews*.

[95] Whitehouse J. D., Friedman N. D., Kirkland K., Richardson W. J., Sexton D. J. (2012). The impact of surgical-site infections following orthopedic surgery at a community hospital and a university hospital: adverse quality of life, excess length of stay, and extra cost. *Infection Control and Hospital Epidemiology*.

[96] Darouiche R. O. (2012). Device-associated infections: a macroproblem that starts with microadherence. *Clinical Infectious Diseases*.

[97] Ren L., Lin X., Tan L., Yang K. (2011). Effect of surface coating on antibacterial behavior of magnesium based metals. *Materials Letters*.

[98] Robinson D. A., Griffith R. W., Shechtman D., Evans R. B., Conzemius M. G. (2010). In vitro antibacterial properties of magnesium metal against *Escherichia coli*, *Pseudomonas aeruginosa* and *Staphylococcus aureus*. *Acta Biomaterialia*.

[99] Zheng L., Li K., Ning C., Sun J. (2021). Study on antibacterial and fluoride-releasing properties of a novel composite resin with fluorine-doped nano-zirconia fillers. *Journal of Dentistry*.

[100] Pollick H. (2018). The role of fluoride in the prevention of tooth decay. *Pediatric Clinics of North America*.

